# Antiosteoporosis Medication Prescriptions After Fragility Fractures

**DOI:** 10.1001/jamanetworkopen.2024.38393

**Published:** 2024-10-09

**Authors:** William K. Silverstein, Sping Wang, Mahnaz Alavinejad, Ania Sarnocinska, Nathan M. Stall, Kamil Malikov, Michael P. Hillmer, Jonathan S. Zipursky

**Affiliations:** 1 Department of Medicine, University of Toronto, Toronto, Ontario, Canada; 2 Division of General Internal Medicine, Sunnybrook Health Sciences Centre, Toronto, Ontario, Canada; 3 Institute of Health Policy, Management, and Evaluation, University of Toronto, Toronto, Ontario, Canada; 4 Ontario Ministry of Health, Toronto, Ontario, Canada; 5 Women’s Age Lab, Women’s College Hospital, Toronto, Ontario, Canada; 6 Division of General Internal Medicine and Geriatrics, Sinai Health System and University Health Network, Toronto, Ontario, Canada; 7 Division of Clinical Pharmacology and Toxicology, Sunnybrook Health Sciences Centre, Toronto, Ontario, Canada

## Abstract

This cohort study assesses prescribing rates for antiosteoporosis medication following a fragility fracture and factors associated with filling a prescription after a fracture among patients in Ontario, Canada.

## Introduction

Pharmacologic treatment for secondary osteoporotic fracture prevention is recommended after an initial fragility fracture of the hip, vertebrae, or pelvis, regardless of bone mineral density, to reduce subsequent fracture risk.^1^ Failing to provide antiosteoporosis medication following a fragility fracture is termed the *osteoporosis care gap*. Contemporary prescribing rates of antiosteoporosis medications after an initial fragility fracture are not well described at the population level. We quantified the osteoporosis care gap in Ontario, Canada, and identified factors associated with filling a prescription for an antiosteoporosis medication.

## Methods

This population-based, retrospective cohort study used data obtained from the Ontario Ministry of Health for all patients aged 66 years and older with an initial fragility fracture of the hip, vertebrae, or pelvis who were admitted to an Ontario hospital from January 1, 2017, to December 31, 2021. Datasets were linked using unique identifiers at Ontario’s Ministry of Health and analyzed from July 2023 to April 2024. We identified fragility fractures using a validated algorithm (eMethods in Supplement 1).^2^ Sunnybrook Health Sciences Centre’s Research Ethics Board approved this study and waived informed consent because deidentified data were used. We followed the STROBE reporting guideline.

Our primary outcome was time to filling a prescription for an antiosteoporosis medication (alendronate, risedronate, etidronate, zoledronic acid, denosumab, raloxifene, or teriparatide) in the 365 days after hospital discharge for fracture. We censored outcomes at 365 days following hospital discharge or at death, whichever came first. We used multivariable Cox proportional hazards regression to model time to prescription fill of an antiosteoporosis medication. Analyses were performed using SAS, version 9.4 (SAS Institute). Additional details on study methods are provided in the eMethods in Supplement 1.

## Results

We identified 69 965 patients aged 66 years and older admitted to hospital with a hip, pelvis, or vertebral fracture. After exclusions, 37 874 patients (54.1%; median [IQR] age, 84 [77-90] years; 26 169 females [69.1%]) were included (Table 1). Overall, 11 853 (31.3%) filled an antiosteoporosis drug prescription in the year after discharge from hospital for fracture. Annual prescription rates were stable during the study interval. Risedronate was the most commonly prescribed antiosteoporosis medication (n = 5677). Factors associated with filling a prescription for an antiosteoporosis medication (Table 2) included female sex (hazard ratio [HR], 1.23; 95% CI, 1.17-1.28), vertebral fracture (HR, 1.30; 95% CI, 1.21-1.41), and discharge to a rehabilitation hospital (HR, 1.64, 95% CI, 1.56-1.72).

**Table 1.** Characteristics of Patients With a Fragility Fracture of the Hip and/or Femur, Pelvis, or Vertebrae

Characteristic	Patients, No. (%) (N = 37 874)
Age, median (IQR), y	84 (77-90)
Age group, y	
66-74	6868 (18.1)
75-84	12 836 (33.9)
≥85	18 170 (48.0)
Sex	
Female	26 169 (69.1)
Male	11 705 (30.9)
Location of residence	
Rural	5129 (13.6)
Urban	32 585 (86.4)
Neighborhood income quintile	
Quintile 1 (lowest)	9888 (26.2)
Quintile 2	8402 (22.3)
Quintile 3	7068 (18.7)
Quintile 4	6378 (16.9)
Quintile 5 (highest)	5965 (15.8)
Admission year	
2017	7435 (19.6)
2018	7590 (20.0)
2019	7791 (20.6)
2020	7357 (19.4)
2021	7701 (20.3)
Discharge destination from hospital	
Home	10 594 (28.0)
Rehabilitation hospital	12 082 (31.9)
Nursing home	6244 (16.5)
Complex continuing care	6354 (16.8)
Other^a^	2600 (6.9)
Fracture type	
Hip and/or femur	30 427 (80.3)
Pelvis	5290 (14.0)
Vertebrae	2157 (5.7)
Rostered to a primary care clinician	32 643 (86.2)
Charlson Comorbidity Index Score^b^	
0	33 712 (89.0)
1	3259 (8.6)
≥2	903 (2.4)
Admission during COVID-19 pandemic^c^	14 426 (38.1)
Drug holiday^d^	5856 (15.5)
Outpatient visit for osteoporosis within 5 y before hospitalization	4163 (11.0)
Internal or geriatric medicine consultation during index hospitalization	19 939 (52.6)
25-Hydroxyvitamin D_3_level measured during hospitalization	4980 (13.1)
Bone mineral density test within 5 y of hospitalization	9466 (25.0)
Diabetes	10 547 (27.8)
Glucocorticoid use	812 (2.1)

^a^ Other included retirement homes and transitional care units.

^b^ Range 0-37, with higher scores indicating higher 10-year mortality.

^c^ March 2020 to December 2021.

^d^ Temporary interruption of drug therapy, defined by receipt of a prescription more than 365 days prior to admission (5-year lookback period).

**Table 2.** Factors Associated With Antiosteoporosis Drug Prescription Fills After a Fragility Fracture of the Hip and/or Femur, Pelvis, or Vertebrae

Factor	Hazard ratio (95% CI)
Age group, y	
66-74	1 [Reference]
75-84	1.06 (1.01-1.12)
≥85	0.84 (0.80-0.89)
Sex	
Female	1.23 (1.17-1.28)
Male	1 [Reference]
Location of residence	
Rural	0.89 (0.84-0.96)
Urban	1 [Reference]
Neighborhood Income Quintile	
1 (lowest)	0.84 (0.78-0.90)
2	0.88 (0.82-0.94)
3	0.96 (0.90-1.03)
4	0.93 (0.87-0.99)
5	1 [Reference]
Admission date	
Before February 2020	1 [Reference]
During COVID-19 pandemic^a^	0.94 (0.91-0.98)
Discharge destination (ref home)	
Home	1 [Reference]
Rehabilitation hospital	1.64 (1.56-1.72)
Nursing home (first time admission)	1.52 (1.39-1.66)
Nursing home (returning to nursing home residence)	1.14 (1.06-1.23)
Complex continuing care	1.44 (1.35-1.52)
Fracture type	
Hip and/or femur	1 [Reference]
Pelvis	0.94 (0.89-0.997)
Vertebrae	1.30 (1.21-1.41)
Rostered to a primary care clinician	1.08 (1.02-1.14)
Charlson Comorbidity Index Score^b^	
0	1 [Reference]
1	0.98 (0.92-1.05)
≥2	0.80 (0.70-0.91)
Drug holiday^c^	1.15 (1.10-1.22)
Outpatient visit for osteoporosis within 5 y before hospitalization	1.06 (0.99-1.12)
Internal or geriatric medicine consultation during index hospitalization	1.11 (1.06-1.15)
25-Hydroxyvitamin D_3_ level measured during index hospitalization	1.40 (1.33-1.47)
Bone mineral density test within 5 y of hospitalization	1.28 (1.23-1.34)
Diabetes	0.95 (0.91-0.99)
Glucocorticoid use within 1 y of hospitalization	1.08 (0.96-1.21)

^a^ March 2020 to December 2021.

^b^ Range 0-37, with higher scores indicating higher 10-year mortality.

^c^ Temporary interruption of drug therapy, defined by receipt of a prescription more than 365 days prior to admission (5-year lookback period).

## Discussion

In this cohort study of nearly 40 000 older adults with an initial fragility fracture between 2017 and 2021, we found that fewer than one-third filled a prescription for antiosteoporosis medication in the year after hospital discharge. Despite well-intentioned systemwide efforts to increase postfracture prescribing,^3^ we found that limited progress has been made in closing the osteoporosis care gap.

Multiple factors are associated with the persistent osteoporosis care gap.^4^ Patient factors include lack of knowledge about osteoporosis, nonadherence to treatment recommendations, and worries about rare drug adverse effects, including atypical femur fractures and osteonecrosis of the jaw. Clinician factors include failure to offer treatment to patients with advanced age and cognitive impairment, limited familiarity with antiosteoporosis medications, and concerns about the adverse implications of early antiresorptive therapy for fracture healing.^5^ Health system factors include unclear jurisdiction for medical management of osteoporosis in hospitalized patients, lack of reimbursement for high-quality osteoporosis care, and a disconnect between acute and primary care.

Study limitations include the inability to evaluate appropriateness of antiosteoporosis medication prescribing, inability to confirm medication adherence because we could only confirm medication dispensation, and limited generalizability of the findings as the study was performed in a Canadian province where patients have universal access to medical care. Overall, our findings underscore the need for renewed and expanded health system efforts to improve postfracture prescribing of antiosteoporosis medications, particularly for patients at highest risk of undertreatment. These efforts could include implementation of fracture liaison services or multifaceted interventions (patient and clinician education, notifications, and reminders), which have the best evidence for improving prescribing rates.^6^

## Notes

Supplement 1.
eMethods.

eReferences

Supplement 2.
Data Sharing Statement
