# Supplementary material for: Antiosteoporosis Medication Prescriptions After Fragility Fractures
Source: JAMA Netw Open. 2024 Oct 9;7(10):e2438393. doi: 10.1001/jamanetworkopen.2024.38393 (PMC11465096; doi:10.1001/jamanetworkopen.2024.38393)
Supplement: Supplement 1. — eMethods. eReferences [file jamanetwopen-e2438393-s001.pdf]

Supplemental Online Content

Silverstein WK, Wang S, Alavinejad M, et al. The osteoporosis care gap—antiosteoporosis medication prescriptions after fragility fractures. *JAMA Netw Open*. 2024;7(10):e2438393. doi:10.1001/jamanetworkopen.2024.38393

eMethods.

eReferences

This supplemental material has been provided by the authors to give readers additional information about their work.

© 2024 Silverstein WK et al. *JAMA Network Open*.

METHODS

ICD-10 codes for identifying fractures based on previously validated algorithms [1].

**Fracture type**	**ICD-10 Code**
Hip	S72.0 - Fracture of neck of femur<br>S72.1 - Pertrochanteric fracture<br>• Intertrochanteric fracture<br>• Trochanteric fracture<br>S72.2 - Subtrochanteric fracture
Vertebral	S22.0 - Fracture of thoracic vertebra, Fracture of thoracic spine NOS<br>S22.1 - Multiple fractures of thoracic spine<br>S32.0 - Fracture of lumbar vertebra, fracture of lumbar spine
Femur	S72.3 - Fracture of shaft of femur<br>S72.4 - Fracture of distal end of femur<br>S72.7 - Multiple fractures of femur<br>S72.8 - Fractures of other parts of femur<br>S72.9 - Fracture of femur, part unspecified<br>• Applicable To Fracture of thigh NOS Fracture of upper leg NOS<br>• Type 1 exclusion for fracture of hip NOS
Pelvis	S32.1 - Fracture of sacrum<br>S32.3 - Fracture of ilium<br>S32.4 - Fracture of acetabulum<br>S32.5 - Fracture of pubis<br>S32.7 - Multiple fractures of lumbar spine and pelvis<br>S32.8 - Fracture of other and unspecified parts of lumbar spine and pelvis<br>• Fracture of:<br>• ischium<br>• lumbosacral spine NOS<br>• pelvis NOS

© 2024 Silverstein WK et al. *JAMA Network Open*.

Exclusion criteria

We excluded patients that filled an anti-osteoporosis medication prescription in the preceding 12 months, had chronic kidney disease, were diagnosed with an atypical femur fracture or osteonecrosis of the jaw in the preceding five years, were diagnosed with a traumatic fracture on index hospitalization, were discharged to a palliative care unit, or died during their index hospitalization. We identified patients with chronic kidney disease[2], atypical femur fractures or osteonecrosis of the jaw[3], or a traumatic fracture[4] using previously validated algorithms.

Primary outcome

The primary outcome was time to prescription fill for an anti-osteoporosis medication in the 365 days after hospital discharge for fracture. Specific medications we examined included alendronate, risedronate, etidronate, zoledronic acid, denosumab, raloxifene, teriparatide. These medications are approved by Health Canada and are included on the Ontario Drug Benefit's database.

Research ethics approval

Sunnybrook Health Sciences Centre's Research Ethics Board approved this study.

© 2024 Silverstein WK et al. *JAMA Network Open*.

eReferences

1. O’Donnell S, Group CCDSS (CCDSS) OW. Use of administrative data for national surveillance of osteoporosis and related fractures in Canada: results from a feasibility study. *Arch Osteoporos*. 2013;8:143. doi:10.1007/s11657-013-0143-2

2. Fleet JL, Dixon SN, Shariff SZ, et al. Detecting chronic kidney disease in population-based administrative databases using an algorithm of hospital encounter and physician claim codes. *BMC Nephrol*. 2013;14(1):81. doi:10.1186/1471-2369-14-81

3. Black DM, Geiger EJ, Eastell R, et al. Atypical Femur Fracture Risk versus Fragility Fracture Prevention with Bisphosphonates. *New England Journal of Medicine*. 2020;383(8):743-753. doi:10.1056/NEJMoa1916525

4. Cui Y, Lix LM, Yang S, Morin SN, Leslie WD. A population-based study of postfracture care in Manitoba, Canada 2000/2001–2014/2015. *Osteoporosis International*. 2019;30(10):2119-2127. doi:10.1007/s00198-019-05074-8

© 2024 Silverstein WK et al. *JAMA Network Open*.
