# Supplementary material for: Antiosteoporosis Medication Prescriptions After Fragility Fractures
Source: JAMA Netw Open. 2024 Oct 9;7(10):e2438393. doi: 10.1001/jamanetworkopen.2024.38393 (PMC11465096; doi:10.1001/jamanetworkopen.2024.38393)
Supplement: Supplement 2. — Data Sharing Statement [file jamanetwopen-e2438393-s002.pdf]

Data Sharing Statement

Silverstein. The Osteoporosis Care Gap—Antiosteoporosis Medication Prescriptions After Fragility Fractures. *JAMA Netw Open*. Published October 09, 2024.
doi:10.1001/jamanetworkopen.2024.38393

Data
Data available: Yes
Data types: Deidentified participant data
How to access data: The dataset from this study is held securely in coded form at the Ontario Ministry of Health (MOH). Legal data sharing agreement with Ontario MOH would be required for access to those who meet prespecified criteria for confidential access. To initiate the process e-mail Kamil.Malikov@ontario.ca. The full analytic plan and underlying code are available from the authors upon request, understanding that the computer programs may rely upon coding templates or macros that are unique to the MOH and are therefore either inaccessible or may require modification
When available: With publication

Supporting Documents
Document types: None

Additional Information
Who can access the data: The dataset from this study is held securely in coded form at the Ontario Ministry of Health (MOH). Legal data sharing agreement with Ontario MOH would be required for access to those who meet prespecified criteria for confidential access. To initiate the process e-mail Kamil.Malikov@ontario.ca. The full analytic plan and underlying code are available from the authors upon request, understanding that the computer programs may rely upon coding templates or macros that are unique to the MOH and are therefore either inaccessible or may require modification
Types of analyses: The dataset from this study is held securely in coded form at the Ontario Ministry of Health (MOH). Legal data sharing agreement with Ontario MOH would be required for access to those who meet prespecified criteria for confidential access. To initiate the process e-mail Kamil.Malikov@ontario.ca. The full analytic plan and underlying code are available from the authors upon request, understanding that the computer programs may rely upon coding templates or macros that are unique to the MOH and are therefore either inaccessible or may require modification
Mechanisms of data availability: The dataset from this study is held securely in coded form at the Ontario Ministry of Health (MOH). Legal data sharing agreement with Ontario MOH would be required for access to those who meet prespecified criteria for confidential access. To initiate the process e-mail Kamil.Malikov@ontario.ca. The full analytic plan and underlying code are available from the authors upon request, understanding that the computer programs may rely upon coding templates or macros that are unique to the MOH and are therefore either inaccessible or may require modification
